# Proximal tibial morphology and risk of posterior tibial cortex impingement in patients with AA-sized Oxford unicompartmental knee arthroplasty tibial implants

**DOI:** 10.1186/s13018-020-01900-6

**Published:** 2020-09-03

**Authors:** Jiun-Ran Charng, Alvin Chao-Yu Chen, Yi-Shen Chan, Kuo Yao Hsu, Chen-Te Wu

**Affiliations:** 1 Department of Orthopedic Surgery, Chang Gung Memorial Hospital, No. 5, Fu-Hsin Street, Kweishan, Taoyuan Taiwan; 2 Bone and Joint Research Center, Chang Gung Memorial Hospital, No. 5, Fu-Hsin Street, Kweishan, Taoyuan Taiwan; 3 School of Medicine, Chang Gung University, No. 259, Wenhua 1st Road, Kweishan, Taoyuan Taiwan; 4 Department of Medical Imaging and Intervention, Chang Gung Memorial Hospital, No. 5, Fu-Hsin Street, Kweishan, Taoyuan Taiwan

**Keywords:** Oxford, Unicompartmental knee, Keel impingement, Proximal tibial, Morphology, Asian population

## Abstract

**Background:**

In cases of Oxford unicompartmental knee arthroplasty (UKA), an increase in anteroposterior and medial-lateral length is usually disproportional when comparing AA and A-sized tibial components. Asynchronous increments may cause tibial keel impingement leading to complications.

**Methods:**

Radiographic measurements were performed in five patients with AA-sized tibial implants. The posterior cortex of proximal tibia had two angles recorded as ∠ M1 and ∠ M2. The minimum distance between the tibial component keel and outer margin of the posterior tibial cortex (mDKC) was measured, and the correlation between the preoperative posterior slope angle (PSA), ∠ M1, and mDKC was analyzed.

**Results:**

All patients showed an acceptable component positioning. Only one patient had an mDKC of < 4 mm that fulfilled the criteria for the posterior tibial cortex at risk. The patient had an increased PSA and ∠ M1 compared to other patients. A negative correlation was found between preoperative PSA and mDKC (*r* = − 0.935, *p* = 0.0193); and ∠ M1 and mDKC (*r* = − 0.969, *p* = 0.0032). However, no stem tip pain, periprosthetic fracture, or component loosening were observed.

**Conclusions:**

The distance between the tibial keel and posterior tibial cortex was reduced in AA-sized patients with a large PSA and ∠M1; therefore, the risk of the tibial cortex injury should be considered.

## Background

Although Oxford unicompartmental arthroplasty (UKA) is one of the most successful surgical options for medial compartment osteoarthritis, catastrophic complications can occur and can be difficult to manage. Of these, periprosthetic fracture and sinking of tibial components have been recognized as the most devastating complications. Precise bone cuts and adequately sized components are key to good results and prevent unnecessarily weakened tibia and uneven stress distribution [1].

During Oxford UKA, the tibial component overhang of 3 mm or more can cause soft tissue irritation and can severely compromise the outcome. Conversely, a tibial component underhang increases the risk of component subsidence and loosening [1]. An optimal match between tibial component and resected tibial surface results in flush edges with the cortical bone, which is the key factor for long-term good results.

Currently, there are a range of sizes of tibial implants in the use for different sized patients. From the smallest to largest, the tibial plates are sized as AA to F. The anteroposterior length (A/P) and medial-lateral width (M/L) of tibial plates and the increase in increments for the length of keel (k A/P) are shown in Table 1. All the differently sized tibial implants have the same thickness plate (2.95 mm), depth (9 mm), and thickness (2.75 mm) of the keel.

**Table 1:** Oxford UKA tibial component size

	AA	A	B	C	D	E	F
M/L^a^ (mm)	24.00	26.00	26.20	28.00	29.80	31.55	33.40
A/P^b^ (mm)	45.39	45.39	48.58	51.79	55.00	58.20	60.90
k A/P^c^ (mm)	28	28	30	33	35	38	40

All differently sized tibial implants have the same plate thickness (2.95 mm), depth (9 mm), and thickness (2.75 mm) of the keel

^a^medial-lateral width of tibial plates

^b^anteroposterior length of tibial plates

^c^anteroposterior length of the keel

It has been recently noted that having keels of the same depth and length can be problematic for people of short stature and may result in an unnecessarily weakened tibia. Both, AA and A-sized tibial components differ in the M/L width (size AA: 24 mm; size A: 26 mm) but have fixed A/P length (45.39 mm). A mismatch in component A/P and M/L increments may result in a relatively posterior located keel in the AA-sized patients, which might increase the risk of injury to the posterior tibial cortex. Oxford UKA tibial implants are generally produced to fit the physique of Caucasians, and many studies have demonstrated that the prostheses designed for Caucasian patients are not suitable for Asian patients. Generally, the size of the tibia in Taiwanese people is relatively smaller than that in Caucasians; hence, smaller sized components are more frequently used. In addition, the Asian population has different tibial intramedullary canal features and proximal tibia morphology compared to Caucasians [2–6]. We, therefore, conducted this study to identify the risk factors for posterior cortex injury associated with AA-sized tibial implants and potential measures for the prevention of complications.

## Materials and methods

### Patients and study design

All the patients receiving Oxford UKA by a single experienced surgeon (KYH) from 2018 to 2019 were retrospectively analyzed in the study group. During this period, five patients with AA-sized tibial implants were identified. All the patients were primary cases without any history of infection, fracture, dislocation, or previous surgery and did not have an extra articular deformity or severe bone loss. No anatomical distortion of the tibial metaphysis was found. The age of the patients at the time of surgery was 71.6 ± 6.8 years, and the body mass index was 26.7 ± 2.1 kg/m^2^. Preoperatively, the coronal plane alignment on standard weight-bearing anteroposterior (AP) radiographs was 6.3° ± 3.9° in valgus, with a range of motion of 5.7° ± 6.1° in extension, and 138.2° ± 13.8° in flexion. All the five patients were females with a preoperative diagnosis of isolated anteromedial compartment osteoarthritis. Full-thickness lateral compartment cartilage and intact medial collateral ligament were checked by valgus stress views, and the function of the anterior cruciate ligament was analyzed by the presence of anteromedial osteoarthritis on lateral plain films; all of which were further confirmed by the addition of preoperative knee MRI.

### Preoperative assessment of tibial posterior slope angle

Preoperative MRI was chosen for the measurements of the posterior slope angle due to better reproducibility than plain radiographs. Based on the sagittal cuts of the preoperative MRI, the images with the widest anteroposterior distance of the medial compartment were selected to choose the measurement film section. The preoperative posterior slope angle (PSA) was defined as the angle between the perpendicular line of the tibial intramedullary canal axis and the line connecting the anterior and posterior borders of the medial tibial plateau [7].

### Postoperative assessment of the tibial component position

Postoperative CT was arranged 2 weeks after the arthroplasty due to better measurement reproducibility than plain radiographs [8] and less metal artifact noise from the tibial components. A 640-row multi-slice CT system (Canon Aquilion One Genesis Edition, Canon Medical Systems, Otawara, Japan) was used to perform the scans. The patients were asked to be in a supine position and kept their knees in fully extended positions with the patella facing upwards. The CT scans included the following parameters: slice thickness 1 mm, tube voltage 120 kV, and tube current 80 mA. All the datasets were processed by SEMAR (single-energy metal artifact reduction) reconstruction and then imported into a dedicated analysis workstation (Vitrea version 6.3; Vital Images, Minneapolis, MN, USA).

**Fig. 1:**
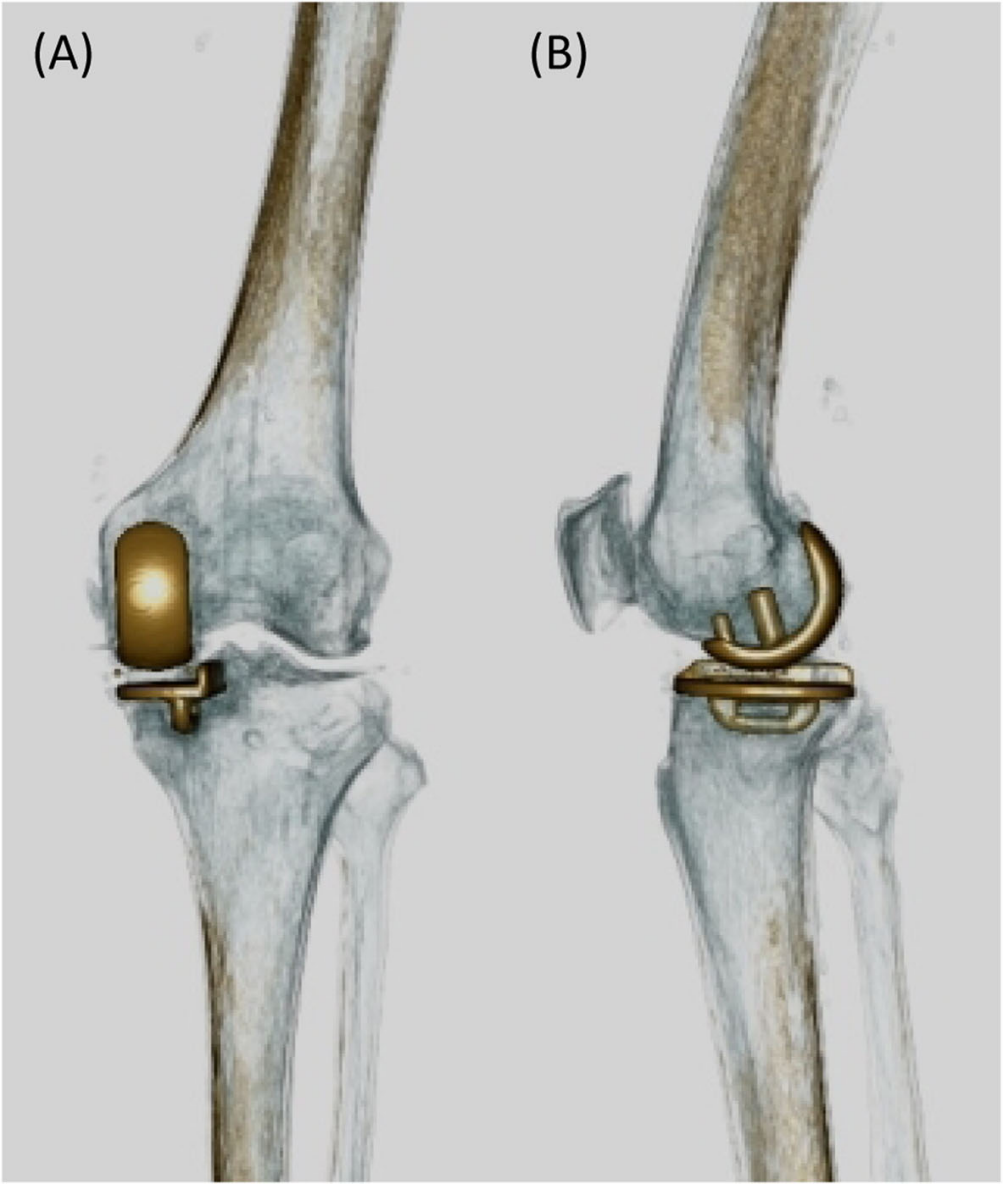
Postoperative 3D CT for assessment of the tibial component. **a** The image shows evenly distributed bilateral condyles and was selected as the representative image for the measurement film section for the AP view. **b** The image shows completely overlapping outlines of the lateral and medial femoral condyles and was selected for the measurement of the lateral view

AP and lateral views from postoperative 3D CT were assessed for coronal and sagittal positions of the tibial component. Images with evenly distributed bilateral condyles were selected for the measurement film section for AP view; while the images with completely overlapping outlines of the lateral and medial femoral condyles were selected for measurement of lateral view (Fig. 1).

The assessment for acceptable tibial implant positioning was performed according to the criteria described in the manufacturer’s surgical technique guide (Oxford Partial Knee Microplasty Instrumentation Surgical Technique). Verification of the tibial component position within the manufacturer’s suggested criteria is a critical parameter to rule out component malposition due to surgical errors. In this measurement, the values of the tibial component varus alignment, posterior slope, and overhang were considered to be positive. Two other parameters not mentioned in the manufacturer’s criteria may also reflect tibial components adequate position and size: the mediolateral positioning and the component rotation.

### Mediolateral position

**Fig. 2:**
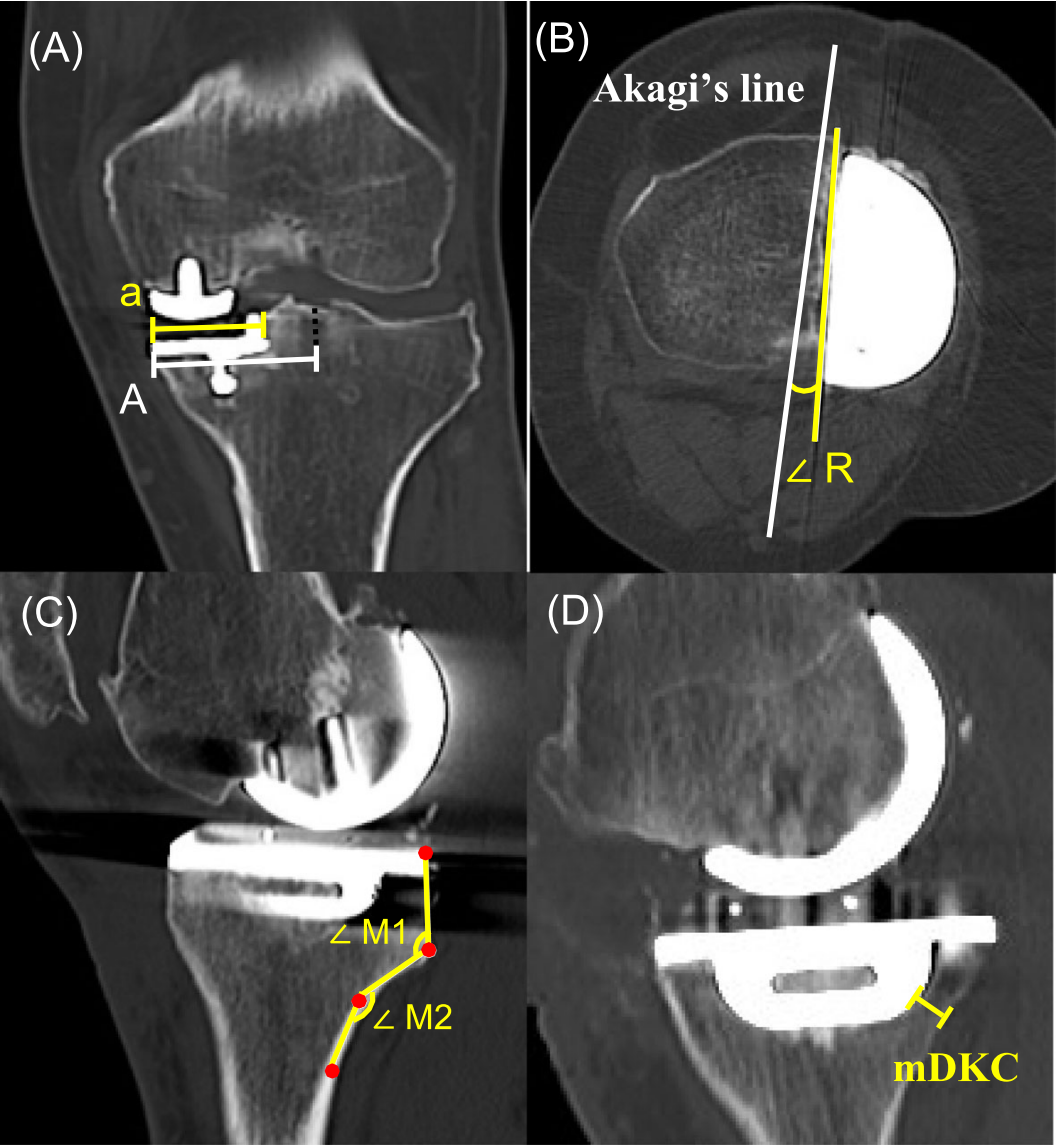
Radiographic measurement of tibial component position. **a** The mediolateral tibial component position was measured and is represented as ratio a/A, where, “a” is the distance from the medial edge of the proximal tibia to the lateral wall of the tibial component and “A” is the distance from the medial edge of the proximal tibia to the tibial axis. **b** The axial alignment of the tibial component was assessed in terms of ∠ R; the angle between a line tangential to the lateral wall of the tibial component and Akagi’s line. Values indicative of external rotation of the tibial component relative to Akagi’s line were considered to be positive. Akagi line is referred to a line connecting the middle of the posterior cruciate ligament and the medial border of the patellar tendon attachment. **c** The posterior cortex of the proximal tibia had two obvious angles with the 90° rotated “Z”-shape. From proximal to distal, the angles were known as the first and second angles, respectively, and were recorded as ∠ M1 and ∠ M2, respectively. All the angles were measured and expressed as acute angles for statistical convenience. **d** The minimum distance between the tibial component keel and the outer margin of the posterior tibial cortex was recorded as mDKC

The mediolateral tibial component position was measured as the ratio a/A, where “a” is the distance from the medial edge of the proximal tibia to the lateral wall of the tibial component, and “A” is the total distance from the medial edge of the proximal tibia to the tibial axis [9]. Smaller values of the mediolateral position indicate a more medial implantation of the tibial component (Fig. 2). Excessive medial implantation suggests inaccurate sizing, and a lateralized vertical cut with A-sized tibial component might be a better option.

### Component rotation

Axial alignment of the tibial component was assessed as the angle R (∠ R) between the line tangential to the lateral wall of the tibial component and Akagi’s line. Akagi et al. [10] noted that in Asian people, the anteroposterior axis of the rotational alignment of the tibial tray is parallel to the line connecting the middle of the posterior cruciate ligament and the medial border of the patellar tendon attachment. The Akagi’s line is considered the most reliable and widely used rotational reference of the tibia [11, 12]. Values indicative of the external rotation of the tibial component relative to Akagi’s line were considered to be positive (Fig. 2).

### Proximal tibia morphology

The postoperative posterior slope angle (post PSA) was defined as the angle between the perpendicular line of the tibial intramedullary canal axis and a line parallel to the tibial component surface. In the sagittal plane, the posterior cortex of the proximal tibia was clearly displayed [13]. The posterior cortex of the proximal tibia had 2 obvious angles with the 90° rotated “Z” shape. The posterior cortex of the proximal tibia can be divided into three segments according to the structure and functional differences: the tibial condyle, metaphysic transition zone, and proximal tibial shaft. From proximal to distal side, the angles between the first and second segments, as well as the second and third segments, were known as the first and second angles, and were recorded as ∠ M1 and ∠ M2 (Fig. 2). All the angles were measured and expressed as acute angles for statistical convenience.

The minimum distance between the tibial component keel and the outer margin of the posterior tibial cortex (mDKC) was measured (Fig. 2). The cortex of the proximal tibia was approximately 2–4 mm in thickness and tended to be relatively thicker in the posterior tibia plateau [14]. Therefore, patients with the mDKC < 4 mm were categorized into a high-risk group for injury to the posterior tibial cortex [15]. Finally, the correlation between preoperative PSA and mDKC was analyzed.

### Radiographic evaluation

The images were transferred digitally to a picture archiving and communication system (PACS). The radiographic magnification of all the measurements was corrected using the PACS ruler and the assessment was performed on a 21.3-inch monitor (Totoku CCL358) in portrait mode using the GE Centricity software. The minimum differences that the software could detect were 0.1° in angle and 0.1 mm in length. All the measurements and calculations were conducted by a single-qualified observer (JRC), who had accepted the measurement training. All the parameters were measured three times on different days by each observer, and the average was taken as the final data. The intra- and interobserver reliabilities of all measurements were assessed using the intraclass correlation coefficient, the values of which were greater than 0.8.

## Results

**Fig. 3:**
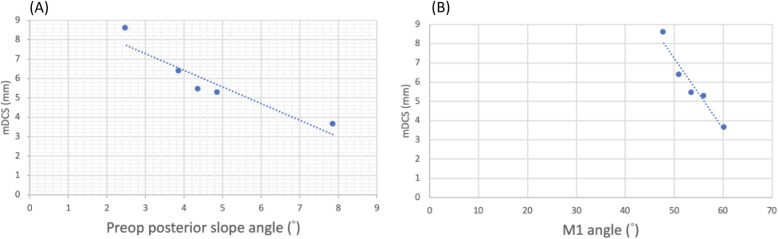
Pearson’s correlation graph shows. **a** Highly negative correlation between the preoperative PSA and mDKC (*r* = − 0.935, *p* = 0.0193). **b** Highly negative correlation between the ∠ M1 and mDKC (*r* = − 0.969, *p* = 0.0032)

Five patients were included (mean age 71.6 ± 6.8 years; all female; 3 right knees, 2 left knees; mean body mass index (BMI): 26.7 ± 2.1 kg/m^2^) in this study. They were followed at an average of 10.2 months (range 6–18 months). Correct tibial cuts and component positioning in all five patients were verified (Table 2). All the patients had measurements within the radiographic criteria of acceptable component positioning. Only one patient had an mDKC < 4 mm which fulfilled the criterion for the posterior tibial cortex at risk. The patient showed an increased PSA and ∠ M1 compared to others. A negative correlation was found between the preoperative PSA and mDKC (*r* = − 0.935, *p* = 0.0193); the ∠ M1 and mDKC (*r* = − 0.969, *p* = 0.0032) (Fig. 3). As per the actual occurrence of complication associated with injury of the posterior tibial cortex, no patient’s knees showed stem tip pain, periprosthetic fracture, or component loosening occurred during the follow-up period.

**Table 2:** Radiographic results

	Patient 1	Patient 2	Patient 3	Patient 4	Patient 5
Varus/valgus (°)	2.8°	2.3°	3.5°	2.6°	3.4°
Preop PSA^a^ (°)	7.9°	2.5°	4.9°	4.4°	3.9°
Postop PSA^a^ (°)	3.9°	3.1°	3.6°	3.1°	3.4°
Medial fit (mm)	1.26	1.55	1.34	1.2	0.23
Posterior fit (mm)	0	0.2	0.1	0	0
Anterior fit (mm)	− 1.27	1.16	0	0	− 1.3
Lateral fit (mm)	0	0.2	0	0.1	0
Depth of tibial saw cut (mm)	2.3	2.4	3.1	2.6	2.5
a/A	0.74	0.71	0.68	0.71	0.67
∠ R (°)	3.4	3.5	2.7	4.3	4.4
∠ M1 (°)	60.4	47.8	56.3	53.7	51.2
∠ M2 (°)	46.4	42.1	45.7	38.8	43.2
mDKC^b^ (mm)	3.58^*^	8.54	5.19	5.41	6.31

All five patients fulfilled the correct component positioning criteria of Oxford Partial Knee Microplasty Instrumentation Surgical Technique

^a^PSA: angle between the perpendicular line of the tibial intramedullary canal axis and the line connective anterior and posterior borders of the medial tibial plateau preoperatively or the line parallel to the tibial component surface postoperatively

^b^mDKC: minimum distance between the tibial component keel and the outer margin of the posterior tibial cortex

^*^< 4 mm: posterior tibial cortex at risk

## Discussion

The most important finding of the present study is that the risk of injury to the posterior tibial cortex might be high when using the current AA-sized prosthesis in patients with specific proximal tibial morphology. The mDKC tended to decrease with an increase in preoperative PSA and ∠ M1.

Accordingly, larger PSA and ∠ M1 are associated with the proximity of the posterior tibial cortex with the tibial keel and an additional risk of impingement. The aim of tibial cutting was to achieve a posterior slope angle close to native status, or at least within 5° of error. Therefore, we consider our postoperative PSA within normal limits and not a result of technical error. Therefore, we believe that greater attention should be paid to prevent posterior tibial cortical injury when the current AA-sized prosthetics are implanted in knees with an increased preoperative PSA and ∠ M1.

The risks of posterior tibial cortical injury can be linked to the bony geometric characteristics of Asian people. Asian knees are smaller in sizes [4, 5], and smaller knee tends to have a larger aspect ratio of the proximal tibia (the ratio of mediolateral/anteroposterior length) [5, 6]. The current AA-sized implants with the same anteroposterior length as A-sized implants are bound to have increased risk of posterior cortex impingement.

Several studies in the western population have revealed that the axis of the tibial shaft is located anteromedial to the center of the tibial plateau [16, 17]. Tang et al. [4] noted that the correlation between the tibial shaft axis to the center of the tibial plateau was different in the Chinese population, in which the axis of the tibial shaft is typically located anterolateral to the center of the tibial plateau. Similar findings were noted in previous studies involving Asian subjects [18, 19]. Nagamine et al. [18] studied 133 Japanese patients and noted that the tibial shaft axis was typically located lateral to the mechanical axis. In a study of 246 Korean patients, the tibial shaft axis was typically located lateral to the mechanical axis on AP radiographs [19]. After total knee arthroplasty with a medially offset tibial keel, contact between the keel tip and the medial tibial cortex was observed in some Asian patients [19]. It is, therefore, reasonable to presume that the possibility of cortex impingement can also occur in Oxford UKA which locates in the medial compartment, especially in Asian patients.

**Fig. 4:**
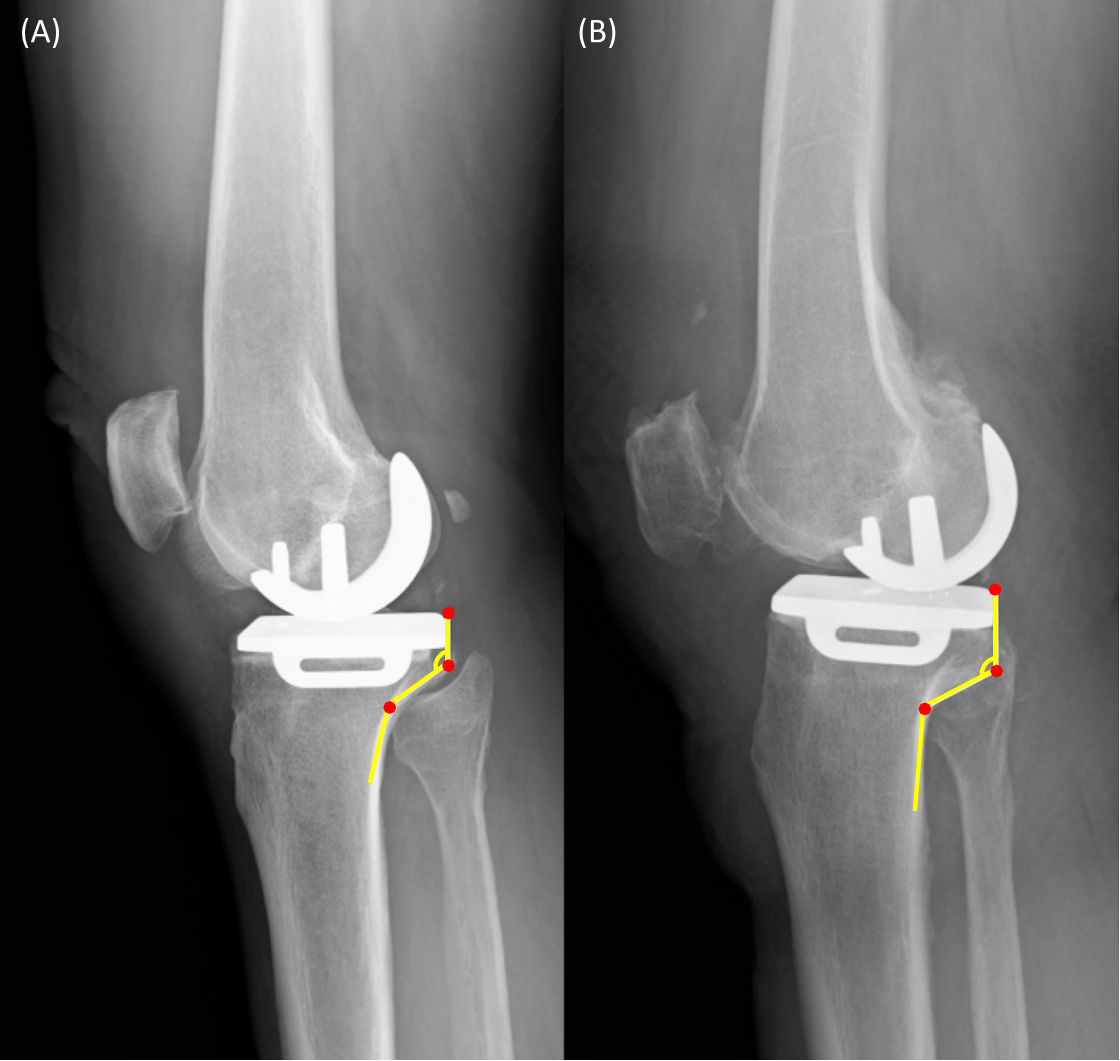
Comparison between the tibial keel and posterior tibial cortex distances among patients with different proximal tibial morphology. **a** A larger ∠ M1 with a smaller distance between the keel and posterior cortex. **b** A smaller ∠ M1 with a larger distance between the keel and posterior cortex

There is a significant variation in the morphology of the posterior column of the proximal tibia (Fig. 4). The cortex tappers posteriorly and forms two obvious angles in the longitudinal direction with the 90° rotated “Z”-shape [13]. We believe that the most possible site of impingement occurs along the posteromedial cortex, while different morphologies of the posterior cortex can affect its distance from the tibial component keel. In our study, an increase in ∠ M1 is associated with a decrease in mDKC. The ∠ M1 represents the most proximal inner turn of the posterior cortex which correlates with the metaphysic transition zone between the posterior condyle and proximal tibial shaft. A larger ∠ M1 reflects a more prominent and protruding type of “posteromedial column,” a component in the four-column classification of tibial plateau fractures [20, 21]. The tibial intramedullary canal is located further anterior to the center of the tibial plateau, and the undersurface of the posteromedial column is in a greater proximity to the tibial keel in patients with increased ∠M1.

**Fig. 5:**
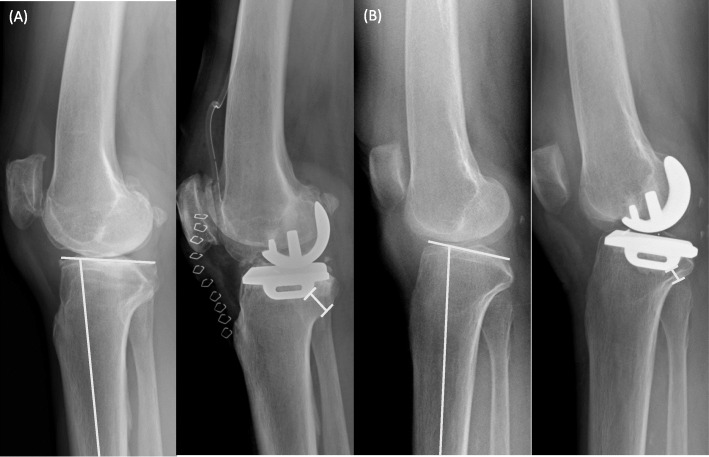
Comparison of the minimal distance between the tibial keel and posterior tibial cortex in patients with or without a high preoperative posterior slope angle (PSA). **a** A smaller PSA with a larger distance between the keel and posterior cortex. **b** A larger PSA with a smaller distance between the keel and posterior cortex

As previously mentioned, the distance between the axis of the tibial shaft and the center of the tibial plateau is an important factor for tibial keel impingement. In addition to the offset in the mediolateral direction, an increased anteroposterior offset may further bring the tibial keel in proximity with the posterior cortex. The tibial intramedullary canal is located anterior to the center of the tibial plateau in Asian compared to Caucasian [2, 3]. Furthermore, Asians tend to have an increased posterior slope angle as compared to Caucasians, which further increases the risk of posterior cortex impingement [22, 23]. The posterior slope angle has a large influence on anteroposterior tibial plateau and shaft offset. The larger the posterior tibial slope, the tibial metaphysis tilts posterior and the center of the knee tends to be located more posterior to an anatomical axis of the tibia. A greater change in the posterior slope angle makes the tibial component keel closer to the tibial posterior cortex to gain the ideal postoperative PSA of 0–7° for oxford UKA (Fig. 5).

No coronal or rotational malposition of tibial components was found in the patients. However, there may be an additional risk of damage to the posterior tibial cortex when the tibial component keel is placed in excessive valgus or external rotation. Inoue et al. [24] reported a remarkably increased stress concentration in the medial and posterior tibial cortex when the tibial component is placed in valgus inclination. Placement of the tibial component with a large valgus inclination should be avoided when considering the risk of medial tibial condyle fractures; especially in patients with AA-sized components. Kamenaga et al. [25] reported that external rotational error of tibial sagittal cuts can affect tibial coverage and keel line. An excessive external rotational error of approximately 10° may decrease the bone mass supporting the tibial components where the body weight load is concentrated and, therefore, is a major risk factor for fracture in Oxford UKA. The rotational position of the tibial component also influences the clinical outcome as shown by Kamenaga et al. [9, 11]. A trend towards poor outcome was observed when the tibial component was placed at a higher angle of external rotation [11]. Bearing impingement with the lateral wall along with poorer knee flexion angles may occur if the tibial component is placed too medially or in pronounced external rotation [9]. Previous studies suggest that placing the tibial component in a more varus and internally rotated position prevents medial condyle fracture and improves clinical outcomes. This is even more pronounced in patients with AA-sized tibial components. Therefore, care should be taken to prevent excessive valgus and external rotation of the tibial components, which shifts the tip of the keel more medial and closer to the tapered edges of the posterior-medial cortex.

After recognizing the risk of injury to the posterior tibial cortex, some of the surgical practices for the current small-sized prosthesis were performed more carefully in our hospital. When dealing with short-statured patients, every effort was made to ensure the most lateralized vertical tibial cut to pursue an A-sized tibial component. If further lateralization was limited and the anterior cruciate ligament was at risk, slight internal rotating of the vertical cuts was made to provide another 2 mm of mediolateral space. We did not encounter another AA-sized component after following the new practice. If an AA-sized tibia component is inevitable, we recommend placing it in a more varus, anterior, and internal rotated position to place the keel away from the posteromedial cortex.

To the best of our knowledge, no previous study has analyzed the radiographic parameters related to the injury risk to the tibial posterior cortex following Oxford UKA using the current AA prosthesis in an Asian population. In this study, we evaluated the radiographic results after Oxford UKA using the AA-sized tibia component and found that an increased preoperative PSA and ∠ M1 is associated with the injury risk to the posterior tibial cortex when using the current prosthesis. The current design has the keel depth fixed for all size tibial implants. We believe that the keel depth of the tibial component has an important role in fractures at the proximal tibia with UKA, especially in patients with a smaller stature. Decreasing the stem length, depth, or distal tapering might reduce the injury risk to the posterior tibial cortex. We believe that current tibial implants for smaller “AA-sized” people are over-engineered and can be made with shallower or shorter keels.

We recognize the limitations of this study, in particular, the small sample size on which the conclusions are based. More cases are necessary to obtain a precise cut-off value for preoperative PSA and ∠ M1 and to determine patients at risk. We assessed the injury risk from radiographic measurements and evaluated the occurrence of complications after a relatively short-term follow-up. The average 10.2-month follow-up should be sufficient to evaluate the radiographic parameters for the position of the components and the occurrence of stem tip pain and fractures. However, it could be insufficient to investigate the actual occurrence of loosening of the tibial components. Hence, a prospective study with a longer follow-up period is required to address postoperative complications. However, we would like to emphasize on the injury risk to the posterior tibial cortex when using the current prosthesis in our ethnic group or Asian people.

## Conclusion

The risk of injuring the posterior tibial cortex might increase in Asian people when using the current-manufactured prosthesis AA sizing of Oxford UKA. Therefore, we believe that greater attention should be paid to prevent posterior tibial cortical injury when the AA-sized prostheses are implanted in knees with a larger preoperative PSA and ∠ M1. There may be a risk of damage to the tibial cortex in patients with such morphology when the keel of the tibial component is placed in excessive valgus inclination, external rotation, medialization, and posterior overhang.

## Data Availability

All data generated or analyzed during this study are included in this published article.

## Abbreviations

UKA: Unicompartmental knee arthroplasty
PSA: Preoperative posterior slope angle
AP: Anteroposterior
CT: Computed tomography
PACS: Picture archiving and communication system
BMI: Body mass index
